# Isolation and Genomic Characterization of SARS-CoV-2 Omicron Variant Obtained from Human Clinical Specimens

**DOI:** 10.3390/v14030461

**Published:** 2022-02-24

**Authors:** Pragya D. Yadav, Nivedita Gupta, Varsha Potdar, Sreelekshmy Mohandas, Rima R. Sahay, Prasad Sarkale, Anita M. Shete, Alpana Razdan, Deepak Y. Patil, Dimpal A. Nyayanit, Yash Joshi, Savita Patil, Triparna Majumdar, Hitesh Dighe, Bharti Malhotra, Jayanthi Shastri, Priya Abraham

**Affiliations:** 1 Indian Council of Medical Research-National Institute of Virology, Pune 411021, India; potdarvarsha9@gmail.com (V.P.); sreelekshmy88@gmail.com (S.M.); dr.rima.sahay@gmail.com (R.R.S.); prasadsarkale123@rediffmail.com (P.S.); anitaaich2008@gmail.com (A.M.S.); patildeepak8@gmail.com (D.Y.P.); nyayanit.dimpal@gmail.com (D.A.N.); yashjos1401@gmail.com (Y.J.); varshapatil111@yahoo.com (S.P.); triparna.majumdar@gmail.com (T.M.); dighehitesh48@gmail.com (H.D.); priya.abraham@icmr.gov.in (P.A.); 2 Indian Council of Medical Research, V. Ramalingaswami Bhawan, P.O. Box 4911, Ansari Nagar, New Delhi 110029, India; drguptanivedita@gmail.com; 3 Genestrings Diagnostic Centre Pvt. Ltd., 3, MMTC, Geetanjali Enclave, New Delhi 110017, India; alpana.razdan@genestrings.co.in; 4 Viral Research and Diagnostic Laboratory, Sawai Man Singh Medical College, Jaipur 302004, India; drbhartimalhotra@gmail.com; 5 Viral Research and Diagnostic Laboratory, Kasturba Hospital for Infectious Diseases, Mumbai 400011, India; jsshstri@gmail.com

**Keywords:** SARS-CoV-2, Omicron, B.1.1.529, isolation, Syrian hamster, cell culture

## Abstract

Due to the failure of virus isolation of the Omicron variant in Vero CCL-81 from the clinical specimens of COVID-19 cases, an initial in vivo and subsequent in vitro approach was utilized for the isolation of the virus. A total of 74 oropharyngeal/nasopharyngeal specimens were collected from SARS-CoV-2 positive international travellers and a contact case at Delhi and Mumbai, India. All the specimens were sequenced using next-generation sequencing and simultaneously inoculated onto Vero CCL-81 cells for virus isolation. Subsequently, two omicron positive specimens were inoculated into Syrian hamsters for two passages. The initial passage of the positive hamster specimens was inoculated onto Vero CCL-81 cells. The clinical specimens, hamster specimens, and Vero CCL-81 passages were sequenced to assess the mutational changes in different host species. The replication of the Omicron variant in hamsters was confirmed with the presence of a high viral load in nasal turbinate and lung specimens of both passages. The successful isolation of the virus from hamster specimens with Vero CCL-81 was observed with cytopathic effect in infected cells and high viral load in the cell suspension. The genome analysis revealed the presence of L212C mutation, Tyrosine 69 deletion, and C25000T nucleotide change in spike gene of hamster passage sequences and an absence of V17I mutation in E gene in hamster passage sequences, unlike human clinical specimen and Vero CCL-81 passages. No change was observed in the furin cleavage site in any of the specimen sequences, suggesting intact pathogenicity of the virus isolate. Our data demonstrated successful isolation of the Omicron variant with the in vivo method first followed by in vitro method. The virus isolate could be used in the future to explore different aspects of the Omicron variant.

## 1. Introduction

The ability of SARS-CoV-2 to rapidly mutate has been the biggest challenge the world has faced while responding to the COVID-19 pandemic. Speculation and the anticipation of SARS-CoV-2 becoming endemic have come to an end with the recent emergence of a heavily mutated variant, Omicron (B.1.1.529). The fifth variant of concern (VOC) was first reported by South Africa followed by Botswana and Hong Kong in November 2021 [1]. The variant has now spread to all six continents within a few weeks, with India reporting thousands of cases [2]. Omicron has posed a serious public health concern due to the mutations/deletions associated with increased binding affinity to ACE2 (S:Q498R and S:N501Y), increased transmissibility (S:H655Y, S:N679K, and S:P681H), increased viral load (N:R203K and N:G204R), innate immune evasion (ORF1a:L3674-, ORF1a:S3675-, and ORF1a:G3676), and S-gene target failure (S:H69-) [3].

The global scientific community is investing enormous effort to determine the susceptibility, transmissibility, immune escape, and severity associated with Omicron. Such an emergency demands the availability of Omicron virus isolate. Many variants of SARS-CoV-2 have been isolated successfully utilizing Vero, Huh7, and human airway epithelial cells. Various cell lines such as Vero CCL-81, Vero SLAM, MA104, BGM, and Caco-2 have also supported the replication of SARS-CoV-2 [4]. Recently conducted studies revealed that Omicron uses cell surface TMPRSS2 less effectively, which results in a decrease in the ability of syncytia formation [5,6]. Recently, Shuai et al. also demonstrated reduced replication of Omicron variant in Calu3 and Caco2 cells. This could be because Omicron could not effectively use transmembrane serine protease 2 (TMPRSS2) compared to wild type and other variants [7]. Another study revealed that Omicron could infect the ACE2-expressing cells, although it did not infect H1299 cells which suggests the significance of ACE-2 in Omicron infection [8]. However, there are still very limited studies that have reported the isolation of SARS-CoV-2 Omicron variant. Here, we report the isolation and characterization of the Omicron variant from clinical specimens of two COVID-19 cases using the Syrian hamster model and Vero CCL-81 cells.

## 2. Materials and Methods

### 2.1. Clinical Specimens

The oropharyngeal/nasopharyngeal swabs of SARS-CoV-2 positive (E gene: Ct value range 17–30) international travellers (*n* = 73) and contact case (*n* = 1) from Delhi and Mumbai, India were collected during 10 October to 13 December 2021. These specimens were transported under cold chain to ICMR-National Institute of Virology, Pune, India.

### 2.2. In Vitro Virus Isolation Approach

All the specimens were inoculated onto monolayers of Vero CCL-81 cells which was maintained in Eagle’s minimum essential medium (MEM; Gibco, Scotland, UK) supplemented with 10 per cent foetal bovine serum (FBS) (HiMedia, Mumbai, India), penicillin (100 U/mL) and streptomycin (100 mg/mL). Likewise, 100 μL was inoculated onto 24-well cell culture monolayers of Vero CCL-81, before growth medium was decanted. The cells were incubated for one hour at 37 °C to allow virus adsorption, with rocking every 10 min for uniform virus distribution. After incubation, the inoculums specimen was removed and the cells were washed with 1X phosphate-buffered saline (PBS). The MEM supplemented with two per cent FBS was added to each well. The cultures were incubated further in five per cent CO_2_ incubator at 37 °C and observed daily for cytopathic effect (CPE) under an inverted microscope (Nikon, Eclipse Ti, Tokyo, Japan). Cellular morphological changes were recorded using a camera (Nikon, Tokyo, Japan). Cultures that showed CPE on PID-5 were centrifuged at 4815× *g* for 10 min at 4 °C; the supernatants were processed immediately or stored at −86 °C. Further, those that showed CPE were grown in T-25 flasks at P-2 and titration was performed after serial dilution. Tissue culture infective dose 50 per cent (TCID50) values were calculated by the Reed and Muench method. The cells were examined microscopically for cellular morphological changes following inoculation [9].

### 2.3. In Vivo Virus Isolation Approach

With subsequent efforts, two Omicron positive specimens [MCL-21-H-11828 (group-A) Ct: 20 and MCL-21-H-12521/1 (group-B); Ct: 25] characterized by whole genome sequencing were selected for in vivo virus isolation. The Syrian hamsters (8–10 weeks old) were anesthetized using the Isoflurane. A hundred microlitre volumes of each specimen were inoculated through the intranasal route into two hamsters. The nasal turbinate (NT), lung specimens of passage 1 (P-1) were further passaged into new batch of hamsters (P-2). The hamsters were sacrificed and NT, lung specimens were collected on the 3rd day post-infection (DPI). NT and lung tissues (20% suspension) homogenates were prepared in sterile Minimum Essential Medium (MEM; Gibco, Waltham, MA, USA) using a homogenizer and were screened using rRT-PCR [10]. SARS-CoV-2 positive NT and lung homogenized suspension of hamsters (P-1 and P-2) were further inoculated onto Vero CCL-81 cells.

### 2.4. Next-Generation Sequencing and Data Analysis

The clinical specimens, hamster specimens, and virus isolates were sequenced using next-generation sequencing with the Illumina MiniSeq Machine. Briefly, the ribosomal RNA depletion was performed using Nebnext rRNA depletion kit (human/mouse/rat) followed by cDNA synthesis using the first strand and second synthesis kit. The RNA libraries were prepared using TruSeq Stranded Total RNA library preparation kit. The amplified RNA libraries were quantified, normalized and loaded on the Illumina sequencing platform [11]. Reference-based mapping was performed to retrieve the complete genome sequence of the clinical specimens, hamster specimens and cell culture specimens using the CLC genome workbench V 20.0.4 and submitted to the public repository, i.e., GISAID. A phylogenetic tree was generated using the MEGA software version 7. The nucleotide variation of the sequences analysed in the study was generated using the highlighter plot (https://www.hiv.lanl.gov/cgi-bin/HIGHLIGHT/highlighter.cgi, accessed on 28 December 2021) <0.05 were considered to be statistically significant.

## 3. Results

### 3.1. Characterization of the Circulating SARS-CoV-2 Variants

The complete genome (>98%) were retrieved from 61 clinical specimens. Forty six sequences belonged to Delta derivatives (Delta (*n* = 2), B.1.617.2.106 (*n* = 1), B.1.617.2.120 (*n* = 1), B.1.617.2.122 (*n* = 2), B.1.617.2.125 (*n* = 3), B.1.617.2.4 (*n* = 2), B.1.617.2.43 (*n* = 1), B.1.617.2.6 (*n* = 2), B.1.617.2.85 (*n* = 2), AY.122 (*n* = 11), AY.125 (*n* = 4), AY.36 (*n* = 1), AY.4 (*n* = 4), AY.4.2.1 (*n* = 1), AY.43 (*n* = 4), AY.46 (*n* = 1), AY.5.4 (*n* = 1), AY.89 (*n* = 1), AY.9.2 (*n* = 2)) while 15 were identified as B.1.1.529. Among the 15 Omicron cases (median age:34 years, male:6/female:9), 14 were international travellers from United Arab Emirates, South Africa, Nigeria, Tanzania, France, UK, Germany, The Netherlands, and one contact case from Mumbai. Thirteen were asymptomatic while two had mild cold, cough, and body ache.

### 3.2. Virus Isolation with Syrian Hamster Was Successful than on Vero CCL-81 Cells

An attempt of isolation using Vero CCL-81 yielded 10 Delta derivatives (AY.125 (*n* = 4), AY.122 (*n* = 2), AY.89 (*n* = 1), AY.46 (*n* = 1), AY.4 (*n* = 1), AY.4.2.1 (*n* = 1)). The Omicron positive clinical specimens were blindly passaged thrice; however, replicating virus could not be detected using rRT-PCR and no CPE was observed [5,6].

**Figure 1:**
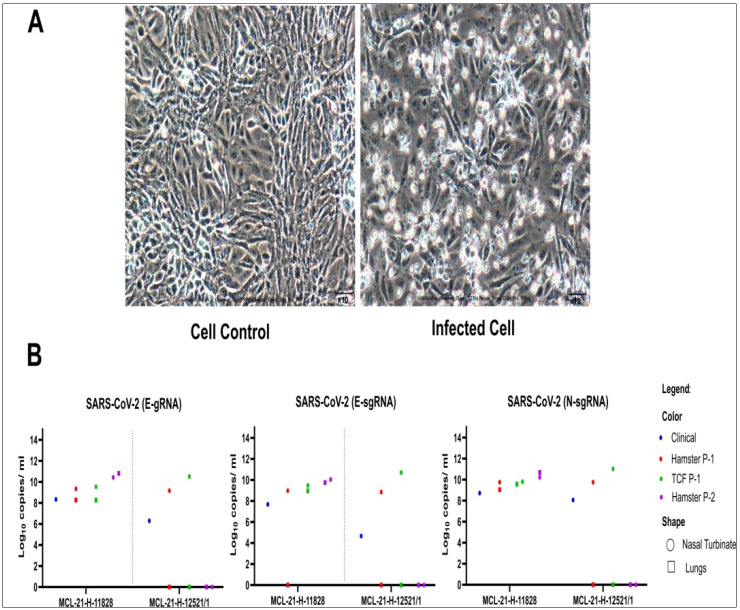
Omicron variant virus isolation in the Vero CCL-81 cells: (**A**) Vero CCL-81 cell control (Left) and Omicron infected Vero CCL-81 cell (Right) at passage 2, PID-4. (**B**) The viral load of the SARS-CoV-2 in human clinical samples, hamster specimens at passage 1 and 2 and Vero CCL-81 at passage 1 culture. The genomic RNA and subgenomic RNA load for the E gene along with N gene from nasal turbinate and lungs of two different clinical specimens.

Subsequently, in vivo isolation of the virus from clinical specimens demonstrated active replication of the virus in both the hamsters of group-A, while group-B hamsters showed replication in NT but not in lungs (Figure 1A). NT and lungs of hamsters group-A had genomic viral RNA (gRNA) copies of 2.0–2.2 × 10^9^ and 2.3 × 10^5^–1.9 × 10^8^ per mL, respectively (Figure 1B), while NT of hamsters from the group-B had gRNA copies of 1.5 × 10^8^–8.3 × 10^9^. The P-1 NT and lung specimens of group-A hamsters inoculated in new batch of hamsters (P-2) demonstrated gRNA copies of 2.6 × 10^10^ and 6.6 × 10^10^ in NT and lungs, respectively, on 3rd DPI (Figure 1B).

### 3.3. Subsequent Virus Isolation on Vero CCL-81 Cells

The hamsters NT and lung homogenized specimens (P-1) inoculated onto Vero CCL-81 cells (P-1) showed evidence of cell rounding and detachment from post infection day (PID)-4. Syncytial cells formed large cell masses that increased in size and number as the infection progressed. Enhanced CPE was noted in P-2 at PID-3. Incomplete cell detachment from the tissue culture plate surfaces, rounding and refractive appearance of infected cells was observed (Figure 1A). The hamster NT and lung specimens of group-A showed typical CPE in P-1 and P-2 of Vero CCL-81 with gRNA copies of 3.4 × 10^9^ to 1.9 × 10^10^ and 1.8 × 10^8^ to 1.0 × 10^9^ per mL, respectively. Sub genomic RNA was also detected in isolates obtained from NT specimens of two hamsters (P-1: 2.9 × 10^9^ and 5.0 × 10^10^; P-2: 4.5 × 10^9^ and 1.3 × 10^10^) and lung specimen of one of the hamster (P-1: 1.8 × 10^8^; P-2: 1.1 × 10^9^) (Figure 1B. While only hamster NT (P-1 and P-2) specimen of group-B displayed CPE in Vero CCL-81 with gRNA copies of 3.3 × 10^10^ and 4.8 × 10^10^ (Figure 1B). The day wise titration of the virus isolate demonstrated a titre of 10^5^/mL, 10^6^/mL, 10^5.3^/mL, 10^4.6^/mL, and 10^4.6^/mL at 1 to 5 PID, respectively.

### 3.4. Genomic Analysis of Clinical Specimens and SARS-CoV-2 Isolates

**Figure 2:**
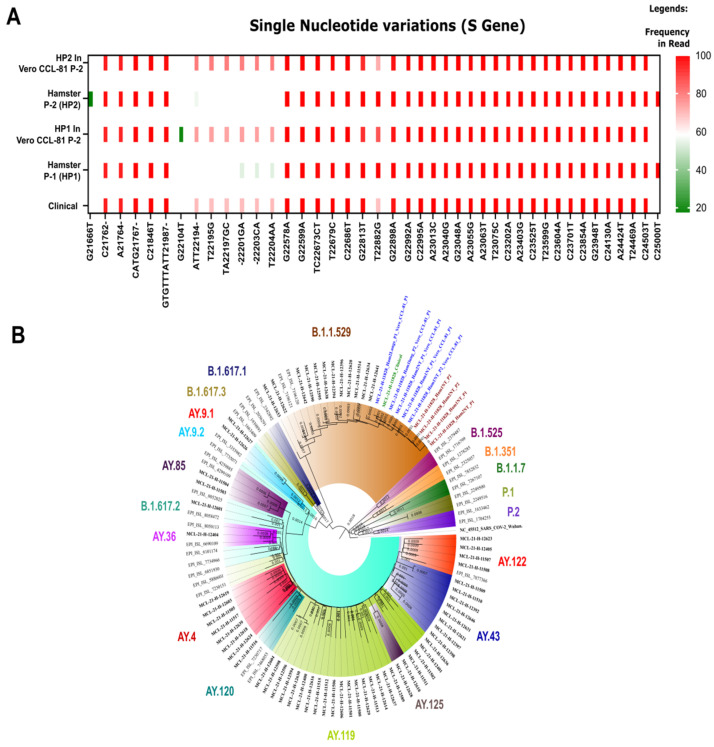
Single nucleotide variations (SNV) and phylogenetic tree of the Omicron clinical and its isolate sequences: (**A**) Single nucleotide variation in the S gene and its frequency in different in vivo and in vitro sequences. The x axis is the SNVs and y axis marks the frequency. (**B**) Neighbour joining tree with a bootstrap replication of 1000 cycles to assess statistical robustness. The sequences retrieved from human clinical specimen, hamsters passages, and cell culture passages are marked in green, brown, and blue boldfaced font, respectively. The sequences of other SARS-CoV-2 variants retrieved in this study highlighted with black boldfaced. The representative SARS-CoV-2 sequences are marked in black regular font.

The molecular characterization of the virus isolates obtained with in vivo and in vitro method confirmed them as Omicron (Figure 2A). The Omicron variants and its isolates clustered together with other representative SARS-CoV-2 sequences (Figure 2B). Single nucleotide variations (SNV) were observed using the CLC genomics Workbench ver. 22.0. The differences were observed in the Omicron sequences from the human clinical sample (GISAID accession number EPI_ISL_8542916), hamster samples (P-1 and P-2) (GISAID accession number EPI_ISL_8542936 and EPI_ISL_8542938), and Vero CCL-81 samples (GISAID accession number EPI_ISL_8542941 and EPI_ISL_8542942). The insertion of AGT nucleotides at genomic position 22,195 was observed in the hamster P-1 sequence and this insertion changed two amino acid encoding codons compared to the human clinical specimens sequence. The first two nucleotide (AG) lay in the first codon while the third nucleotide insertion ‘T’ lay in the other codon. Hence, the reading frames of the codons were changed from ATA to AAGTGC. However, when compared to the reference spike protein of Wuhan sequence, the amino acid at position 211 and 212 were replaced by “KC” instead of “NL”. The tyrosine at 69 amino acid position of spike was lost in the hamster in P-1 and observed in hamster P-2. These mutations need to be further explored which might have facilitated the viral entry and replication in hamster. Further, a nucleotide change of ‘C’ to ‘T’ was observed at position 25,000 only in the hamster sequences.

A gain of an SNV expand was observed at the E gene (G26293A (V17I)) of P-1 Vero CCL-81 isolate, while it was absent in clinical as well as hamster sequences. Further, an SNV was also observed at M gene (C26577G (Q19E)) of hamster P-1 which was not observed in the clinical sample, Vero CCL-81 P-1, or hamster P-2. An in-depth analysis would be required to understand the gain or loss of this specific mutation.

The frequency of the N440K amino acid change was observed in the hamster sequences (100%) and in the human clinical sequences (68.5%). The Vero CCL-81 passage demonstrated the decrease by 86.0% in Vero CCL-81 P-1 of the hamster (P-1) and 71.79% in Vero CCL-81 (P-1) of the hamster (P-2). The variation in the frequency of the N440K in human clinical specimens, hamster specimens, and Vero CCL-81 passages could be due to the adaptive mutations in different host species. A recent study linked this mutation to be associated with higher infectious titre [12]. Further, the studies have reported that the N440K strains have the capability of escaping the neutralization [13].

## 4. Discussion

This study reports the isolation of the Omicron variant using the in vivo followed by in vitro method of virus culture. Until now, there has been no literature available on the isolation of SARS-CoV-2 using initial in vivo and subsequent in vitro approach. Spike region of the virus isolated in hamsters revealed L212C amino acid change along with the C25000T nucleotide change compared to clinical specimens. A percent nucleotide difference of 0.0066% was observed, indicating a minimal change of Omicron sequence in Vero CCL-81 P-1 with hamster P-2. Nonetheless, a recent study conducted by VanBlargan et al. reported that even a single mutation such as R346K might be critical for neutralization escape. The possibility of uncontrolled mutations will be difficult to account [14]. A detailed analysis for further passages with Vero CCL-81 needs to be explored.

Apparently, the sequences of human clinical specimens, hamster specimens, and Vero CCL-81 passages did not show any mutations in the furin cleavage site during sequential passages in different host species. Such virus isolate would retain its pathogenicity as per the original specimen/isolate. The isolated Omicron virus would be useful in new vaccine development, vaccine efficacy studies, pathogenicity studies, antiviral research, and estimation of virus neutralizing titers in vaccinated and recovered individuals.

## Data Availability

All data is available and present either in the publication or in the indicated databases.
